# Conceptual Privacy Framework for Health Information on Wearable Device

**DOI:** 10.1371/journal.pone.0114306

**Published:** 2014-12-05

**Authors:** Seyedmostafa Safavi, Zarina Shukur

**Affiliations:** Unit of Cyber Security, Faculty of Information Science and Technology, Universiti Kebangsaan Malaysia, 43600, Bangi, Malaysia; King Saud University, Kingdom of Saudi Arabia, Saudi Arabia

## Abstract

Wearable health tech provides doctors with the ability to remotely supervise their patients' wellness. It also makes it much easier to authorize someone else to take appropriate actions to ensure the person's wellness than ever before. Information Technology may soon change the way medicine is practiced, improving the performance, while reducing the price of healthcare. We analyzed the secrecy demands of wearable devices, including Smartphone, smart watch and their computing techniques, that can soon change the way healthcare is provided. However, before this is adopted in practice, all devices must be equipped with sufficient privacy capabilities related to healthcare service. In this paper, we formulated a new improved conceptual framework for wearable healthcare systems. This framework consists of ten principles and nine checklists, capable of providing complete privacy protection package to wearable device owners. We constructed this framework based on the analysis of existing mobile technology, the results of which are combined with the existing security standards. The approach also incorporates the market share percentage level of every app and its respective OS. This framework is evaluated based on the stringent CIA and HIPAA principles for information security. This evaluation is followed by testing the capability to revoke rights of subjects to access objects and ability to determine the set of available permissions for a particular subject for all models Finally, as the last step, we examine the complexity of the required initial setup.

## Introduction

**Figure 1:**
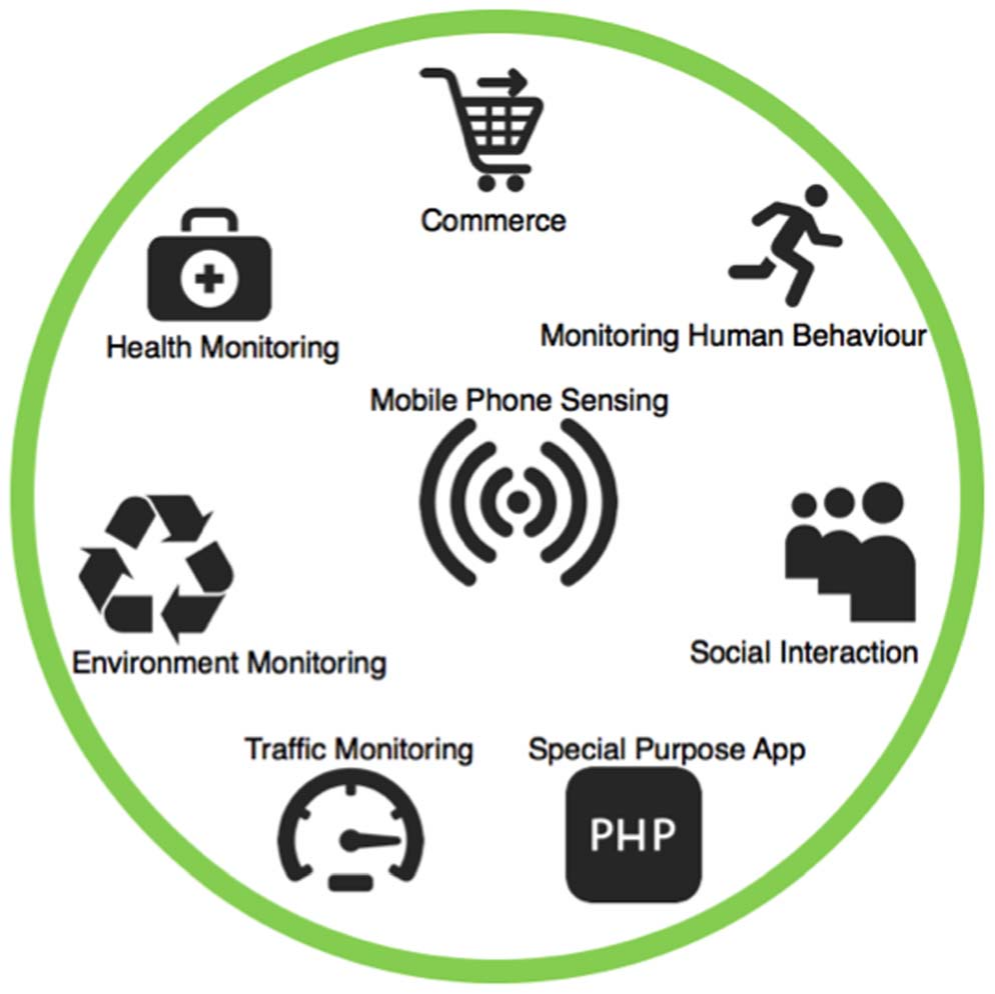
Mobile sensors and area of coverage.

Given that healthcare is one of the most important aspects of not only our lives, but also economy, it is not surprising that it is gaining attention in wearable technology. Healthcare provision via wearable devices brought changes in treatment and examination of patients, as well as research and development in different areas. Given that the advanced communications technology has ensured that most individuals own Smartphones, which are capable of using 3rd party applications and many more devices connected to it, it was logical to find the ways for healthcare to benefit from this trend. This prompted the emergence of a new field called Mobile Health that, according to World Health Organization (WHO), has enabled health checkups to be conducted via Smartphone or some other type of wearable device. It is envisaged that, soon, health management and supervision will be conducted with the help of functions like sound, SMS and MMS, Bluetooth and other services [1] (Figure 1). On the other hand, Google has already made significant progress in this field, by introducing wearable OS called Android Wear and the new types of wearable devices, such as Android watches and Google Glasses (the glasses that can provide their wearer with information and collect it at the same time). Google developers have proposed the idea of inbuilt technology that can use sensors to provide all kinds of information about the user's health [2]. The applications recently announced by Apple (HealthKit), Google (Google Fit) and Samsung (S Health) facilitate health monitoring through sensors that measure a user's heart rate and a gyroscope that calculates a user's movement. This technology and API along it may help providers to collect the most relevant data, because the glasses are something most individuals are used to wearing. Producing devices with such capability could bring much more innovation to the healthcare system. Rapid growth in the number of subscribers to technology, such as cell phone, can ensure connectivity and usability throughout the healthcare system.

According to the report by Wu et al. [3], usage of cellular technology, Smartphones in particular, in the medical field is increasing, since the devices can be used to record patient's medical history, symptoms and details about lifestyle. This information can assist physicians in providing treatment and diagnosis in a safe and effective way. Findings of similar research studies indicate that, with more widespread health IT adoption, we can have an effective means to improve the quality and safety of healthcare [4].

**Figure 2:**
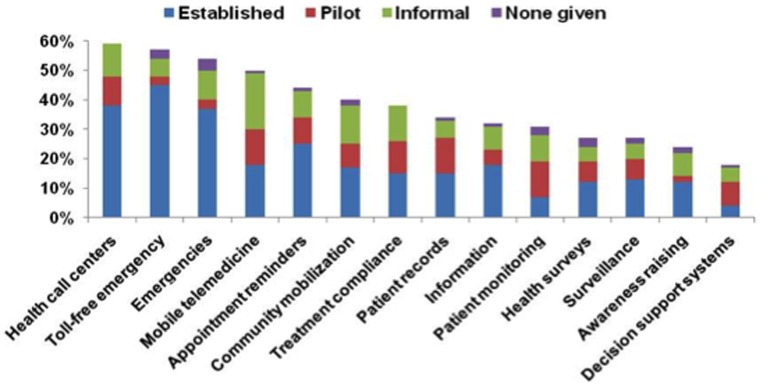
Adoption of mobile health initiatives and phases, globally [1].

However, this innovation also poses an issue of ensuring that protected health information (PHI) is not misused, (protected health information (PHI) is any information about health status, provision of health care, or payment for health care that can be linked to a specific individual). A study recently published by comScore suggests that the number of cell phone subscribers in the US increased from 49 to 76.8 million between 2010 and 2011 [5], [6]. As shown in Figure 2, the demand for portable devices like Smartphone can ensure that they are widely available in healthcare. It is important to note that, if mobile healthcare technology is to be widely utilized, it must be ensured that the client is given full control of the data, even if a different party owns the applications. Thus, issues are likely to rise when one party decides to share information with different companies for profit. Hence, in the context of the healthcare system, it is necessary to apply privacy and safety rules [7].

While wearable healthcare provision devices could bring us better lifestyle and remove the need to regularly visit clinics, it may introduce greater security and privacy issues to our society [8], [9]. Healthcare technology gadgets have to be designed with the aim of improving health, as well as keeping the client out of privacy and secrecy difficulties. Clearly, there is a potential for misuse of health information, which must be addressed.

In this paper, we propose a concise, improved and effective privacy framework for wearable device manufacturers, as well as application developers, capable of providing greater privacy and security to the wearable device owners. The framework consists of two parts—the principles and the checklists. Since the framework is related to the privacy of health information on a wearable device, several critical aspects are considered when constructing the framework.

For creating the checklists, we propose:

- Wearable technology that includes the device technology and its operating system,
- Functionalities of health apps,
- System architecture based on PHR standard.

For formulating the principles, we offer:

- Expanding the existing privacy and security framework related to health information,
- Conducting a healthcare system projects case study.

The proposed framework is subsequently compared with other frameworks based on six CIA and HIPAA principles pertaining to Information Security, namely Confidentiality, Integrity, Availability, Authenticity, Non-repudiation and Information security analysis.

This paper is organized as follows: Section 2 presents a review of the aforementioned aspects, while the proposed framework is described in Section 3. In Section 4, we evaluate the framework, and Section 5 concludes the work.

## Review on Technology and Health Application

### 1. Popular PHR Architectures

Wearable technology, and especially mobile health, can bring advanced supervision to any place at any time without the need for the patient to visit the clinic. As a result, the cost of healthcare can be substantially reduced. To record medical history, two standard formats, Personal Health Record (PHR) and Electronic Health Record (EHR), are used. While PHR is designed by the patient, EHR is designed and supported by the healthcare supplier, such as a clinic or hospital system. Since PHRs contain significant amount of data and must be subjected to stringent privacy rules and controls, they are the main focus of this paper.

**Figure 3:**
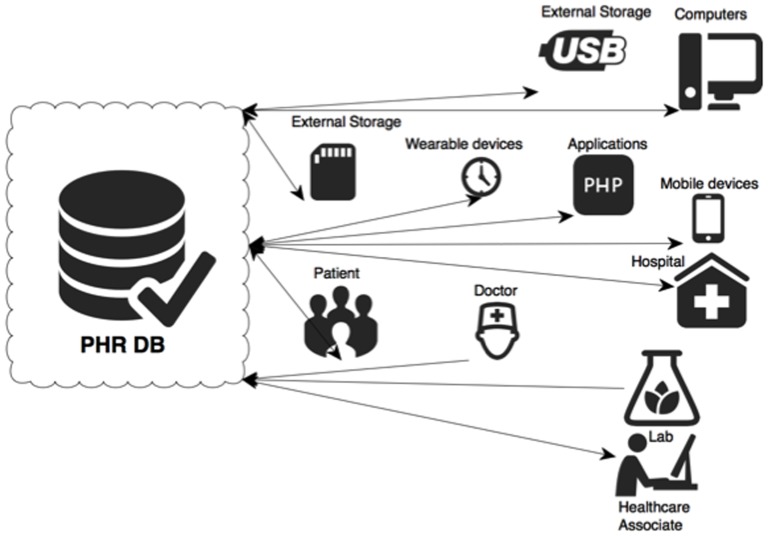
PHR model provided by Microsoft [13].

Presently, Google [10] healthcare service and Microsoft [11], are the two main PHR providers, as shown in Figure 3. Both companies offer applications that permit the users to control their own PHI with the help of Internet. This enables collation of PHI from different sources pertaining to the same person. Moreover, the user can change the elements in the existing record, as well as share the content with family or healthcare providers. Once they have created a Microsoft account, users can control and handle their own or family member's PHR, as well as authorize others to handle their own PHRs at any time and level. As mentioned earlier, as clients can share their own data with whomever they choose, they are effectively accepting the risks associated with the process. PHRs with different types of protection designs [12] indicate that it is likely that how service providers handle the sensitive data and protect the health information will change in the future.

Via Internet, the clients can check the submitted information and visit schedules at anytime, as well as choose a specific doctor to visit. This method involves client sharing the PHI across the PHR model, which may also include receiving data associated with financial claims and lab results by multiple suppliers. Similarly, physicians will receive all the necessary information about the patient across the system.

As the PHR model is based on users holding their data on a central repository, the client must keep and share the entire PHI data via Internet or portable devices. Presently, this includes flash memory or Smartphone, but the number of options is likely to expand in the future. It is essential, however, that these allow the user to add and change fields of database records using the device or a dedicated Internet website.

This model authorizes suitable devices to run processes on user PHR information with specific technology. In case of different vendors providing PHR, a set of standards must be imposed. For example, if a Smartphone health gadget seller supplies a specific application that can record the information received by the same device, it must be recognizable by all other devices involved in the healthcare system and allow the patient to access data with the help of seller's Internet site. In addition, privacy in mobile health in organizations should be using the same client-centric PHRs. Presently, many service providers can provide cellular connectivity. While this reduces the price of technology and expands its usage, it poses much greater risk to security and privacy.

### 2. Operating System Main Features

**Figure 4:**
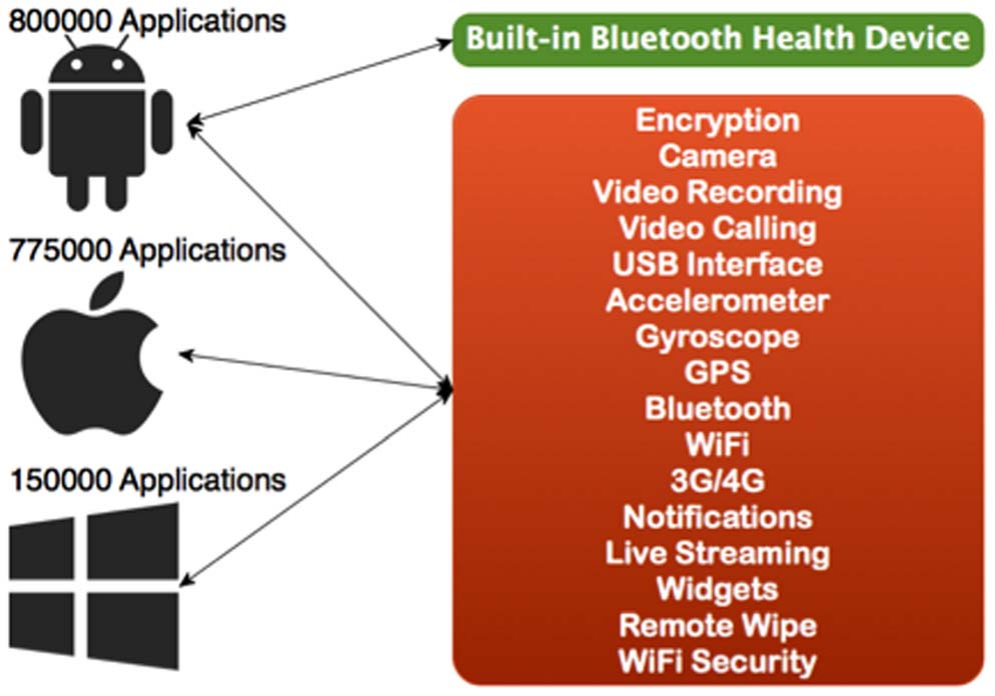
Comparison of Market share percentage and main healthcare features supported by three mobile OS platforms [14].

The most popular platforms are largely similar in terms of allowing the users to receive useful applications. However, their operating systems are typically incompatible. As shown in Figure 4, ideally the platform should be able handle all types of health care devices, with the focus on wearable gadgets that always stay with the user.

The Android Operating System designer and developer (Google company) is focusing on health system since many users use the device and would like to benefit from it in terms of improving their health and fitness.

As shown in Figure 4, the operating system needs to support features that can help applications to connect to the wearable device and have hardware that can receive and store data in real time. In case of any issues or noticeable changes in the user's health, the wearable device should automatically start working in emergency mode.

Among the three most widely used operating systems available in the market, only Android has included built-in connectivity to Bluetooth Health Device Profile (HDP) system [15].

### 3. Brief Review on Popular Wearable Devices

As of April 2014, the Unit of Cyber Security in Faculty of Information Science and Technology, Universiti Kebangsaan Malaysia (UKM) has completed analysis of some wearable devices that are still not available to public worldwide, namely Google Glass from Google Company and Galaxy Gear Fit designed and developed for wearable system from Samsung.

**Figure 5:**
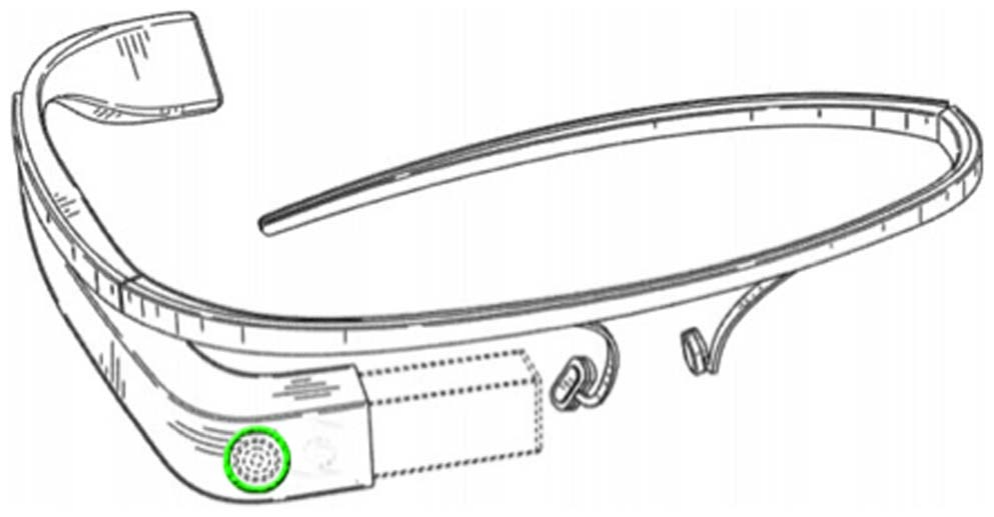
Google Glass design (front view) [1].

As an appointed explorer (the first author) for the latest wearable devices, we found that, with the sensors available in these wearable devices and help of API, healthcare providers can record data and protect the device owner. Presently, they can use the technology to find out the position of the user and record the number of steps taken, along with other fitness measurements, including heart rate measured by the built-in sensor, these devices are incapable of recording and transmitting data, such as location, without connection to a Wi-Fi network or a specific smartphone (Figure 5). This scenario may change with the recently announced Android Wear devices (e.g. Galaxy Live, LG G-Watch R, Moto 360), as well as the Apple Watch, which has a Healthkit system, and the Samsung Gear S, which includes 3G, GPS, and a heart rate monitor.

After proper analysis of devices, we concluded that the mechanism that ensures data security is presently lacking, which must be changed in later versions. Data protection is crucial for widespread use of these devices as a means of keeping daily checks of user's health and lifestyle. Thus, this point is added to our checklist.

### 4. Review of Health Apps

We analyzed the applications made for different Operating Systems in Table 1, based on the platform, functionality and targeted audience of the specified application. In this study, the strategy of selecting applications follows three main criteria: (1) category, which refers to the listed applications categorized under the patients' section, (2) download per application, or the popularity of the application that can lead users to find more useful applications under the first criteria, and (3) the mobile operating system covered by the selected application.

**Table 1:** Applications that we have reviewed.

Application	Platforms	Functions	Main Audience
S Health by Samsung	Android	It can help you achieve your fitness goals by monitoring your fitness levels during workouts and throughout the day. The application will be connected to wearable devices to monitor your health at all times	Patient
Davis's Laboratory and Diagnostic Tests	Android, BlackBerry, iOS, Palm OS, Windows Mobile.	User will receive care before, during, and after the test; RSS receives feeds of clinical lab-product news.	Patient
Pocket Guide to Diagnostic Tests	Android, BlackBerry, iOS, Palm OS, Windows Mobile.	Clinical settings laboratory procedures; diagnostic imaging tests; complex algorithms flowcharts will be ready at all times; laboratory tests; images are color images; and cloud support app.	Patient/Clinics
Labs 360	Android, BlackBerry, iOS, Palm OS, Windows Mobile.	Providing values; cross-reference available; up to date	Healthcare providers
5MCC	Android, BlackBerry, iOS, Palm OS, Windows Mobile	Diagnostic information, algorithms and flowcharts available; drug therapy in every case available.	Doctor/Patient
5MIDC	Android, BlackBerry, iOS, Palm OS, Windows Mobile	Alphabetically listed topics of interest	Patient/Healthcare providers/Doctor
ID Notes	Palm OS	Organs, illnesses and related treatments, different types of indexing.	Doctor/Patient
P.M.I.D	Palm OS, Windows Mobile	Guideline and therapy suggestion; users will be categorized by physical history and therapy specify to the same user.	Patient/Healthcare providers
S.G Antimicrobial Therapy	Palm OS, Windows Mobile, iOS, BlackBerry.	Facility to search; categories available; organized diseases & historical conditions; organized drug info; very efficient navigation.	Doctor/Patient
ePocrates ID	Palm OS, Windows Mobile, iOS, BlackBerry, Android	900 infections, pathogens and drugs recorded in its database; anatomic location and price details by alphabetical categories; ability to record personal notes and information in the database.	Doctor
Mobipocket Reader	BlackBerry, Windows Mobile, Symbian OS, Palm OS	eBooks library; search in the dictionary; customizable display.	Doctor/Patient
J.H.A.Guide	iOS, Android, Palm OS, Windows Mobile, BlackBerry	Includes expert noted information about illnesses; drug lists and interactions; Info on anti-microbial agents;	Doctor
Palm LabDX	Palm OS, Windows CE	Alphabetical tests listed; includes test info; mostly info about testing.	Doctor/Patient
UpToDate	iOS, web-enabled smartphone	Includes 14000 physicians, drug topics and related information; provides search filters to find the information in three categories: adult, pediatric, patient.	Doctor/Patient
EyeChart	iOS	Complete eye charts used by professionals.	Doctor/Patient
Lab Unit Converter	iOS	Conversions and tests, ability to search lab results.	Doctor
Normal Lab Values	iOS	Search reference visualizes labs alphabetically.	Doctor/Patient
DizzyFIX	iOS	Assists treatment of BPPVc	Doctor/Patient
Video Laser Level	iOS	The oculoplastic surgeons can check alignment of canthal position at the time of surgical planning and after the job.	Doctor
eRoentgen Radiology Dx	iOS	Identifies best radiology test for the user; can be searched via diagnosis/symptoms.	Doctor
Antibiotic Dosage Calculator	iOS	Calculation of drug dosages and adjustments based on patient's data t.	Doctor/Patient

After conducting an extensive analysis of applications, we conclude that proper transparency in application, ease of User Interface (UI) use, customer interaction with the system and having moderator and privacy settings are the most important aspects of wearable healthcare applications. Hence, these elements are included into the newly proposed framework.

## The Proposed Framework

The proposed framework considers the client control across the healthcare information, implying the power to share data with a specific organization or a particular person relevant to the subject. At the same time, it is essential to differentiate between level of responses. We expect that the portable and wearable devices, like Smartphone and smart watch, with the help of Internet and application developers, will help rapidly expand the use of healthcare technology [16]. The patient should have the option to share the data monitored and stored by these devices with family members [17], [18], [19], or with 3rd parties. For example, doctors can use the data for diagnosis [20], the insurance system can access the information to provide cover for the user, and researchers can access the records in order to conduct analyses and tests [21]. It is also envisaged that other authorized persons may use this information to design diet, health plan or a training program for the client [22].

**Figure 6:**
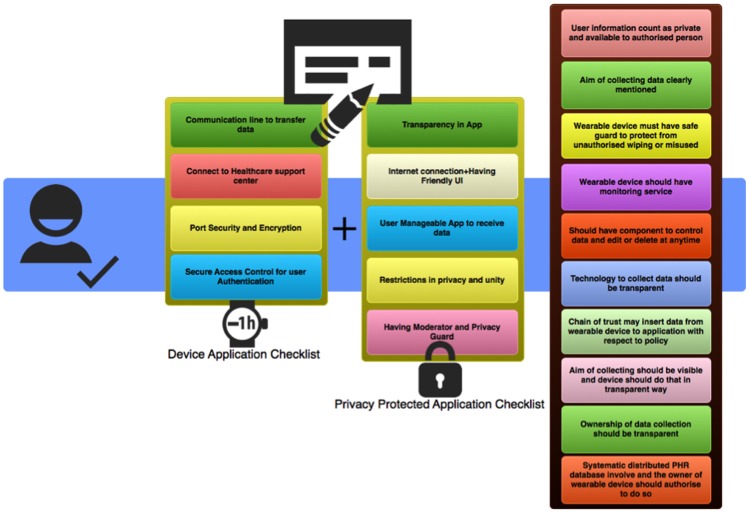
Conceptual framework in Wearable Healthcare System.

The framework depicted in Figure 6 is designed with the aim of improving security and privacy level for the healthcare system owner and it may help device manufacturers to improve privacy checklist. Privacy rules in this framework are sourced from major popular frameworks that have been available since 1999 (ONC, the Markle Foundation, HPP, and CCHIT) [23] and various healthcare system projects (CDT, CF) [24]. In Connecting for Health (CF) project, every device should be capable of transferring data, from forms to the data architecture. In CDT, which is based on using policy scenes of the CF, the focus is on policy papers [25].

### 1. The Principles

We recommend several improvements to both ONC and CF, which are formulated and programmed by various groups of professionals. Since we have focused on information privacy on wearable devices, the CF framework is deemed a more objective framework and, as it is user-centric, it will be covered in the following sections.

The new framework is based on ten essential principles shown in Figure 6.

- **Pr1.** The user-collected information should be treated as private data and only be available to a specific list of authorized persons or companies.
- **Pr2.** The purpose of collecting data should be clearly stated and adhered to.
- **Pr3.** The wearable device should have safeguards to protect the content from unauthorized access or misuse.
- **Pr4.** The device should have monitoring service that can gather information about the application's data collection protocol and the data owner should have the right to monitor, review and stop specific application.
- **Pr5.** Wearable devices should have a component that controls the information stored in the database, as well as allow the data owner to edit or delete content at any time.
- **Pr6.** The technology used to gather the data should be transparent at all times.
- **Pr7.** The device user, or other trusted entities, can insert the personal data from the device to an application specified to collect the data from the owner of the wearable device, while adhering to respected policies.
- **Pr8.** The device should collect data in a transparent way mentioned in a relevant policy and the aim of the collection of information must be completely visible.
- **Pr9.** The ownership of data collection has to be fully transparent and granted solely to the device owner.
- **Pr10.** The systematically distributed PHR database should be involved and the wearable device owner should be able to modify the content at any time.

We acknowledge that wearable healthcare system may provide protection through many different aspects, and the attributes noted above may used by other sectors, not only those related to portable or wearable healthcare system. Since wearable healthcare system is the most likely one to be used, it is a vulnerable service; thus, we may have to adjust remote supervision to control the device. In addition, applications should have the capability to disable remote monitoring and even shut down the device to protect the user's complete PHI package.

### 2. The Checklist for Privacy

This checklist was created after an extensive analysis of the currently available devices capable of supporting the healthcare monitoring system. It is divided to two parts, one pertaining to device protection and the other to data privacy and security.

- Proper device healthcare Application checklist:
  - CL1. Communication line to transfer information.
  - CL2. Direct connection between the user and the support center.
  - CL3. Designed secure port to exchange relevant information between the system and the user.
  - CL4. Secure access control method for authentication of the healthcare user. For example, PIN authentication process, or some system based on biometrics, including an eye focus to open the lock, a retinal scan, voice scan, and so on, could ensure that only the authorized individual can access the data.
- Checklist for privacy protected applications:
  - CLP1. Full transparency in application.
  - CLP2. Internet connected application for Smartphone and easy to use User Interface (UI) for the customer interaction with the system.
  - CLP3. The user-manageable application to receive data.
  - CLP4. Restrictions on information privacy and unity.
  - CLP5. Inclusion of moderator and privacy guard.

### 3. The Improved PHR Model

**Figure 7:**
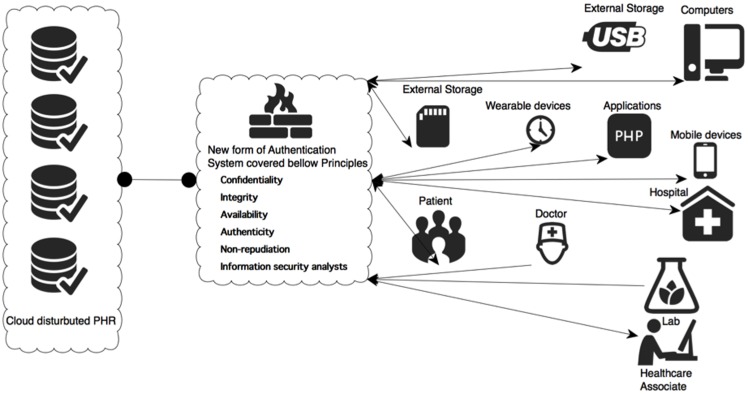
New improved PHR model.

Once the new improved framework was determined, we designed a new and improved PHR model (Figure 7) that would be more secure as well as flexible. Thus, in our view, it can be used to protect the user, while being responsive at all times.

### 4. Framework Evaluation Based on CIA and HIPAA Principles

The evaluation presented here is based on the CIA and HIPAA principles for information security and tried to cover all aspect of privacy and security as well as usability of the model via proper analysis.

As the first step, we tested the ten principles noted above with respect to ensuring better security, as well as providing a user-friendly interface. Thus, we started with flexibility that brings the ability to support frequent changes in policy, and continued with granularity of the process, with respect to the permission levels that can be applied to different objects.

In the second step, we assessed the access control model performance, whereby we tested the authorization complexity of the proposed model. Next, we moved onto analysis that involved modifying access privileges to check if the model is flexible enough. Finally, we tested whether the privileged data is modifiable and, at the same time, attempted to modify the rights of subjects to access objects.

After finishing the analyses noted above, we moved onto testing the capability to revoke rights of subjects to access objects, followed by the ability to determine the set of available permissions for a particular subject for the proposed model. In the final step, we examined the complexity of the required initial setup.

As shown in Figure 7, the requests from different parts of system will be checked by the new framework, which will use the priority settings in order to respond correctly.

### 5. Comparison with major frameworks

This comparison is based on the CIA principles for information security and tries to cover all aspect of the wearable device privacy framework in Table 2. We have divided the table into six principles recommended by CIA, with corresponding keys to the existing and new frameworks. We compare our new framework with Office of the National Coordinator for Health Information Technology Framework (ONC) [23], Health Privacy Project Framework (HPP) [26], Best Practices Framework (BP) [27], Markle Common Framework (CF) [28], The Certification Commission for Healthcare Information Technology Framework (CCHIT) [29].

**Table 2:** Comparison with major frameworks.

CIA Principles	Proposed Framework	Frameworks related to features
**Confidentiality**	Accountability, Access control, Disclosure of data, Treatment Pr9/CL4/CL1	ONC1, ONC2, HPP3, BP3, CF1, CF6, CCHIT4, CCHIT 5, CCHIT 6, CCHIT 7, BP9, CF9, ONC5, HPP2, BP4, BP6, CF4, CF7
**Integrity**	Data quality/integrity, Informed consent Pr2/Pr5	ONC6, ONC7, BP7, BP8, CF6, CF7, ONC4, HPP4, HPP6, BP5, CF1, CF3, CCHIT1, CCHIT3
**Availability**	Access control, Disclosure of data Pr2/Pr1/Pr7/CL2/CL4/CLP2	ONC1, ONC2, HPP3, BP3, CF1, CF6, CCHIT4, CCHIT 5, CCHIT 6, CCHIT 7, ONC5, HPP2, BP4, BP6, CF4, CF7
**Authenticity**	Data quality/integrity, Informed consent, Transparency Pr1/Pr6/Pr4/CL3	ONC6, ONC7, BP7, BP8, CF6, CF7, ONC4, HPP4, HPP6, BP5, CF1, CF3, CCHIT1, CCHIT3, ONC1, ONC 3, ONC 4, HPP 4, HPP 6, BP1, CF2
	Protection safe guard CL4/CLP5/Pr4/Pr10	This feature is not available in any of them
**Non-repudiation**	Transparency, Informed consent, Disclosure of data Pr1/Pr8/Pr2/CLP1	ONC1, ONC 3, ONC 4, HPP4, HPP 6, BP1, CF1, CF2, CCHIT3, ONC4, HPP4, HPP 6, BP5, CF3, CCHIT1, CCHIT3, ONC5, HPP2, BP4, BP6, CF4, CF7
**Information security analysis**	Informed consent, Access control, Accountability CL4/CLP3/CLP4	ONC4, HPP4, HPP 6, BP5, CF1, CF3, CCHIT1, CCHIT3, ONC1, ONC2, HPP3, BP3, CF1, CF6, CCHIT4, CCHIT 5, CCHIT 6, CCHIT 7
	Protection safe guard Pr3/CLP5	This feature is currently not available

### 6. Case Study

We illustrate and evaluate this framework with a wearable privacy awareness system designed for a clinic. For this evaluation, we conduct a case study on a user—a heart surgery patient—to effectively manage his or her situation, including significant variations in his or her blood stress level, frequently elevated blood pressure, and step counts. The doctor advises the patient to subscribe to a health management program designed by the clinic. The patient is also advised to wear a Samsung Wear device that will continuously monitor his activities. The device and application are designed as a wristwatch to prevent any kind of loss or misuse. In the first attempt, the wearable device features pulse waveform and pair-to-pair connection to the patient's mobile system for the authentication of an actual patient. Moreover, if the patient has to move away from the mobile device, he or she has to enter a code in the device to initiate its operation in stand-alone mode (Pr3 and CL4).

During its installation in a smartphone, the application shows the permissions that must be accepted to send information from the mobile and to allow the sensors to continuously provide a report to the installed application (CLP1). After installation, the patient is notified of a carefully designed privacy policy list that is clear, concise, easily understandable, and not too long so as to discourage the patient from simply accepting it without reading it (CLP2, Pr2, Pr8, and Pr6). This privacy policy clearly indicates how information is collected and with whom it will be shared (Pr1). After the patient reads and accepts the privacy policy, the application proceeds to the setup for information collection. This process allows the patient to specify and choose with whom and at what time to share the collected information (Pr9 and CLP4). This selected setting can be changed at any time and is flexible enough to conceal data location when the patient chooses the secrecy option (Pr5). More choices are available for sharing data that can facilitate the level of access to PHI; for example, doctor information and insurance company are not be the same (Pr1). The patient or an authorized person may change the settings and preferences for the specific ID (Pr10 and Pr4) through the mobile, watch, and website interfaces (with username and password).

The device continuously monitors a patient's health and activity and sends encrypted information to the smartphone if paired with a mobile device (CL3). The application in the smartphone is capable of sending information with a set of certified cryptographic keys to a clinic server with a configurable setting of the same application (CL1). It is also capable of verifying that the patient's wearable device is calibrated at all times. The information received by the application in the smartphone is equipped with mobile device capabilities, such as a sensor that determines location (GPS), Wi-Fi address (MAC and localization address), and calendar schedule, to facilitate the effective planning of the patient's health program (Pr4). The application in the wearable device alerts the user to engage in some activity when he or she has free time, such as a notification to exercise by waking or slow jogging.

The wearable device enables the patient to send diet information to the application and the server connected to the device using voice command and camera (Pr7). The device's smart notification reminds the patient to take his or her medicine or sends him or her motivational messages to control his or her diet and activity, but only when the patent's calendar registers free time.

The application in the wearable device, smartphone, and secure website interface with username and password provides the patient information about activities that can help prevent illness (CLP3). The patient's doctor may also monitor and analyze the patient's improvement. However, insurance companies may have access to particular information to keep the patient under insurance rules and regulations (Pr1). In case of device loss or theft, the device is automatically disconnected and wiped remotely after the patient reports loss or the back-end server audit log flags a report. The log is reported to both patient and clinic for further investigation (CLP5).

The same back-end server reports the name of the patient and the time and location collected by the wearable device (CL2). In addition, the updates of the application on the wearable device and smartphone are automatically applied to ensure the security of the device and the remote protection of privacy (CL4 and CLP5).

## Conclusion

In this paper, we improved privacy and security of wearable devices intended for use in healthcare provision by designing a new framework that can work on every wearable operating system. After an extensive analysis of data and frameworks related to the healthcare system, we proposed a different framework that can support new wearable devices, such as Google Glass, Galaxy Gear Fit or Galaxy Wear. The suggested framework incorporates ten essential principles for wearable healthcare systems. In addition, we have proposed a comprehensive checklist that can help both developers and manufacturers to improve the quality of privacy safeguards in their products. We believe that our conceptual framework is one of the most comprehensive concepts available at the time of publishing this paper.

However, we are fully aware that these frameworks cannot be implemented without law enforcement that combines the security and privacy with regulation. Such collaborative effort between technology developers, service providers and law professionals will ensure that healthcare not only becomes cheaper, but also more accessible to all. We hope that this work has contributed to the better understanding of the security protocols.
